# Necessity and least infringement conditions in public health ethics

**DOI:** 10.1007/s11019-017-9775-0

**Published:** 2017-04-29

**Authors:** Timothy Allen, Michael J. Selgelid

**Affiliations:** Monash Bioethics Centre, Monash University, Bldg. 11, Melbourne, VIC 3800 Australia

**Keywords:** Necessity, Least infringement, Least restrictive alternative, Ethics framework, Public health ethics

## Abstract

The influential public health ethics framework proposed by Childress et al. includes five “justificatory conditions,” two of which are “necessity” and “least infringement.” While the framework points to important moral values, we argue it is redundant for it to list both necessity and least infringement because they are logically equivalent. However, it is ambiguous whether Childress et al. would endorse this view, or hold the two conditions distinct. This ambiguity has resulted in confusion in public health ethics discussions citing the Childress et al. framework, as demonstrated by debate between Resnik and Wilson and Dawson. We analyse this debate to resolve these ambiguities. Finally, we argue that the necessity/least infringement principle of the Childress et al. framework applies only in cases in which only one intervention is to be implemented to achieve one specific goal. In other cases, it is not essential to require that only the least infringing intervention be implemented.

## Introduction

Theoretical frameworks to guide normative reasoning in the context of public health often consist of, or include, conditions that public health interventions purportedly must satisfy in order to be ethically acceptable. In this article we analyse two conditions that are included in the influential ethics framework proposed by Childress et al. (2002): “necessity” and “least infringement.” We argue that these two conditions (as described by Childress et al.) are logically equivalent, and that listing both in the framework is thus redundant. However, we argue it is unclear whether or not Childress et al. would endorse this view, or would instead hold the two conditions distinct. This ambiguity of the framework has caused confusion in public health ethics discussions that involve the Childress et al. framework. While debate between Resnik (2010) and Wilson and Dawson (2010) reveals important problems with the necessity and least infringement conditions, for example, it reflects confusion about the meaning and function of the condition(s) in question. We therefore analyse this debate to resolve the ambiguities.

Following this, we compare and contrast the least infringement (and thus necessity) principle as stated by Childress et al. with other least-infringement-type principles in the public health ethics literature. We suggest that least infringement (and thus necessity) is a broad principle that implies corollary principles for each kind of moral cost, such as least restriction of liberty, least infringement of privacy, least infringement of justice, and so on.

Finally, we argue that least infringement (and thus necessity) as stated by Childress et al. involves both an evaluative component and a prescriptive component; and while the evaluative component is self-evidently correct, the prescriptive component may be problematic. This is because the latter might entail that only *one* intervention (i.e., the least infringing alternative) can be ethically justified, even if employment of multiple interventions would bring about more net benefit than a single intervention. The least infringement condition, that is, might sometimes rule out interventions that should be considered ethically justified. The least infringement principle should thus be reserved for cases in which only one intervention is to be implemented to achieve one specific goal.

## The Childress et al. public health ethics framework

The influential public health ethics framework proposed by Childress et al. (2002) includes five conditions that public health interventions must satisfy:

1. effectiveness,
2. proportionality,
3. necessity,
4. least infringement,
5. public justification (173).

Effectiveness requires that “[i]t is essential to show that infringing one or more general moral considerations will probably protect public health” (173). For example, a public health intervention that infringes individual liberties to a high degree, but is expected to be unlikely to succeed in its goals, is most likely ethically impermissible. Proportionality requires that “[i]t is essential to show that the probable public health benefits outweigh the infringed general moral considerations…. All of the positive features and benefits must be balanced against the negative features and effects” (173). Necessity requires that the intervention be “necessary to realize the public health goal that is sought” (173). As the authors put it, “[t]he fact that a policy will infringe a general moral consideration provides a strong moral reason to seek an alternative strategy that is less morally troubling” (173). Least infringement requires that “public health agents should seek to minimize the infringement of general moral considerations” (173). “[W]hen a policy infringes autonomy, public health agents should seek the least restrictive alternative; when it infringes privacy, they should seek the least intrusive alternative” (173), and so on. Public justification states that public health agents have a responsibility to explain and justify to the relevant parties, as far as possible, any infringements of moral considerations that result from public health interventions (173). The relevant parties include those who are affected by the infringement and the general public. This condition helps to ensure transparency, which the authors hold is important because (a) there is a requirement to “treat citizens as equals and with respect by offering moral reasons… for policies that infringe general moral considerations”; and (b) transparency “is essential to creating and maintaining public trust… [and] accountability” (173).

## The necessity and least infringement conditions

At first glance, it seems that there are similarities between the necessity and least infringement conditions of the Childress et al. (2002) framework, which raises questions about what Childress et al. consider the relationship between the two to be. In this section, we analyse the definitions of necessity and least infringement, and then analyse the relation between the two conditions, as intended by Childress et al. Regardless of Childress et al.’s intention, we argue that the two conditions (as stated) must be logically equivalent.

As noted above, the *necessity* condition requires that the public health intervention in question be “necessary to realize the public health goal that is sought,” and that “[t]he fact that a policy will infringe a general moral consideration provides a strong moral reason to seek an alternative strategy that is less morally troubling” (173). In other words, a morally infringing approach cannot be considered necessary to achieve a public health goal if that goal can be achieved by an alternative approach that is less morally problematic. For example,

all other things being equal, a policy that provides incentives for persons with tuberculosis to complete their treatment until cured will have priority over a policy that forcibly detains such persons in order to ensure the completion of treatment (173).

In this case, the policy that provides incentives is morally preferable to the policy that forcibly detains people, because the latter infringes liberty to a higher degree. Thus, assuming that all else is equal, e.g. that the two have equal expected effectiveness and are equal with respect to other moral costs, the policy involving forcible detention does not pass necessity.

There is an ambiguity with the question of whether a policy is “necessary to realize the public health goal that is sought” (Childress et al. 2002, 173). The idea that a policy must be necessary to realise an end could be taken to mean that the policy must be the *only possible way* to realise the end. However, the above example given by Childress et al. reveals that this cannot be their intended requirement of the necessity condition. They state that necessity would prioritise the policy that provides incentives over the policy that forcibly detains people, since—all else being equal—the latter is more morally costly. In this example, the policy that provides incentives is not the *only possible way* to realise the goal, since the forcible detention policy is still an alternative—albeit a morally worse one—that could realise the goal. More generally, it is often the case that there are multiple approaches available to achieve any given public health goal. The necessity condition would be far too stringent if it required that interventions be the one and only one way to realise the end, because it would then rule out intervention in numerous important cases. Instead, the necessity condition as stated/illustrated by Childress et al. simply requires that the costs of a given intervention are necessary in the sense that they cannot be avoided in realising the end—i.e., because there is no less costly alternative that can realise the end.

It is important to highlight that Childress et al. write “all other things being equal” (173), meaning that the necessity condition is supposed to apply only when evaluating interventions that are *equally effective*. All else being equal, if there are two interventions that are equally effective, and one is more morally costly than the other, then it seems self-evidently true that the least morally costly approach is morally preferable. The principle thus appears to be correct. However, the necessity condition, as stated by Childress et al., does not provide guidance regarding how to choose between two interventions that have different levels of effectiveness in achieving the public health goal. If A is more morally costly than B, but A is more effective than B, then the necessity condition does not determine which intervention is morally preferable.

The *least infringement* condition states:

Even when a proposed policy satisfies the first three justificatory conditions—that is, it is effective, proportionate, and essential in realizing the goal of public health—public health agents should seek to minimize the infringement of general moral considerations. For instance, when a policy infringes autonomy, public health agents should seek the least restrictive alternative; when it infringes privacy, they should seek the least intrusive alternative; and when it infringes confidentiality, they should disclose only the amount and kind of information needed, and only to those necessary, to realize the goal. The justificatory condition of least infringement could plausibly be interpreted as a corollary of necessity—for instance, a proposed coercive measure must be necessary in degree as well as kind (173).^1^

Based on the definitions of necessity and least infringement given by Childress et al., the two conditions appear to be logically equivalent.^2^ If an intervention passes the necessity condition, it is because there is no (equally effective) alternative act that is *less infringing* of moral considerations. Because passing necessity entails there is no less costly alternative that can realise the end, an intervention that passes necessity also passes least infringement. That said, it is unclear from the quoted discussion if Childress et al. themselves intend for necessity and least infringement to be logically equivalent, or if they are instead supposed to be distinct in some way.

### The intended relation between necessity and least infringement

If Childress et al. consider necessity and least infringement to be different principles, there seem to be two possibilities for the intended relation between them: either least infringement is a corollary of necessity, or it is not. As noted above, Childress et al. say that “[t]he justificatory condition of least infringement *could plausibly be* interpreted as a corollary of necessity” (173, emphasis added). Whether or not the authors actually intend for least infringement to be a corollary of necessity is an important question because it relates to the logical relation between the two conditions. A corollary is a proposition that logically follows from a previous proposition. Thus, on the ‘corollary approach’ to necessity and least infringement, as we call it, least infringement would follow from necessity. However, even if we assume that Childress et al. would endorse the corollary approach, it is still not clear precisely what sort of corollary relation they have in mind. For example, is least infringement (LI) logically equivalent to necessity (N)—i.e., N implies LI, and LI implies N? Or, rather than logical equivalence, is least infringement merely an implication of necessity—i.e., N implies LI, but LI does not imply N? Either way, satisfying necessity would be sufficient to satisfy least infringement.

If Childress et al. do intend to take the corollary approach to necessity and least infringement, it is unclear why they include least infringement as an *additional* condition to necessity. One possibility is that they include least infringement as a corollary condition simply to make it clear that it *is* a corollary of necessity. Listing corollaries in this way may be appropriate and helpful when said corollaries are not obvious, such as in the case of mathematical theorems entailing corollaries that may be difficult to discover. However, listing least infringement as a corollary condition of necessity does not seem appropriate or helpful in this case because it seems obviously and self-evidently true that least infringement is logically equivalent to necessity (given the way the principles are defined/illustrated by Childress et al.). Another possibility is that Childress et al. have been influenced by the seminal Siracusa Principles—which includes both a “necessity” principle and a version of the least infringement principle. In short, principle 10 of the Siracusa Principles requires that a given intervention be “necessary,” meaning that, inter alia, the intervention responds to a pressing need and is proportionate to its aim; and principle 11 requires that any given intervention uses “no more restrictive means than are required” for the achievement of its aim (UN Commission on Human Rights 1984, 3). Despite sounding similar, the necessity and least infringement conditions of the Childress et al. framework clearly pose different requirements than the two Siracusa principles. Moreover, the two Siracusa principles are not logically equivalent, or even corollaries, while the two Childress et al. conditions are clearly logically equivalent.

The other interpretation of Childress et al.’s position is that they do *not* intend for least infringement to be a corollary of necessity [despite their suggestion that it “could plausibly be interpreted” (173) as such]. On this interpretation, satisfying necessity would not be sufficient to satisfy least infringement, and thus an intervention could (theoretically) pass necessity and fail least infringement. Though Childress et al. do not explicitly address this issue, they make it sound like necessity and least infringement are supposed to be logically distinct conditions that pose separate requirements. Recall their statement that:

Even when a proposed policy satisfies the first three justificatory conditions—that is, it is effective, proportionate, and essential in realizing the goal of public health—public health agents should seek to minimize the infringement of general moral considerations (173).

The authors thus clearly give the impression that the requirement to minimize the infringement of general moral considerations (i.e., the least infringement condition) is separate from the requirement to show that the costs of the intervention cannot be avoided in realising the public health goal (i.e., the necessity condition). Since Childress et al. appear to consider necessity and least infringement as distinct conditions that each apply separately when evaluating interventions, it seems most likely that they do not intend for least infringement to be a corollary of necessity—despite referring to this approach as “plausible.” One possibility that Childress et al. may have in mind is that necessity and least infringement are distinct in the following way: necessity requires that interventions be the least morally costly *kind* of intervention (compatible with realising the public health goal that is sought)—e.g., a tax is arguably a less morally costly kind of intervention than a ban—while least infringement is an additional step requiring that the intervention of the kind in question infringes moral considerations to the least *degree* possible (compatible with realising the public health goal that is sought). Call this interpretation the *kind–degree distinction* approach. It is unclear whether or not Childress et al. would endorse this interpretation, though it is suggested by their statement that a plausible interpretation of the requirements posed by necessity and least infringement is that “a proposed coercive measure must be necessary in *degree* as well as in *kind*” (173, emphasis added). However, aside from this statement, they do not address the relation between necessity and least infringement in detail—nor do they make further appeal to a kind–degree distinction.

Regardless of whether or not Childress et al. would endorse the kind–degree distinction, we argue that it cannot be the correct approach due to two distinct problems. First, it is not clear that differences in kind can be kept distinct from differences in degree in this context. To illustrate, it may be thought that a tax is a different kind of intervention than a ban. Yet, a tax that is high enough—say, a 1,000,000% tax on sugary beverages—is effectively equivalent to a ban, since most individuals could not afford a $30,000 beverage. Perhaps taxes and bans tend to be different kinds of strategies, but there are cases where there is overlap, i.e. where a tax is so restrictive that it, in effect, constitutes a ban.

Even if it turns out that we can plausibly distinguish differences in kind from differences in degree in this context, there are counterexamples to the idea that an intervention belonging to a more infringing *kind* cannot be the least infringing *particular act*/*intervention* when all variables are considered (and when all else is equal). Even if bans tend to be more morally infringing than taxes, the opposite may be true in some cases. For example, suppose a 20% sales tax on sugary beverages over 0.5 litres is found to be more effective at preventing obesity if it targets only the lowest socioeconomic (SES) areas of society, given that obesity rates are higher in those areas (hypothetically). Suppose this tax would be equally effective at preventing obesity as a ban on such beverages that applies across the country and affects all individuals equally. Even so, the tax is arguably more infringing of justice than the ban since the former places a disproportionate burden on the worst off-groups of society. It is thus plausible to think that the *ban* is the less morally infringing act, all things considered, despite being a more infringing *kind* of strategy than the tax. The relevant question therefore is not whether an intervention is the least infringing alternative both in kind and in degree, but instead whether it is the least infringing *particular act*/*intervention*, all things considered (insofar as least infringement is a legitimate ethical principle).

## Resnik’s employment of the Childress et al. framework

Debates in the public health ethics literature involving the Childress et al. framework—and, more specifically, involving the necessity and least infringement conditions—are often characterised by a lack of clarity about the meaning and/or function of the conditions (which may be understandable given some of the ambiguities of the framework). Authors have been criticised for misusing the conditions. For example, Resnik (2010) is criticised for his employment of the conditions in an evaluation of the ethical implications of trans fat (TF) bans, which Resnik reports have been implemented in several areas in the US and in Puerto Rico in order to improve public health. Resnik adopts Childress et al.’s (2002) justificatory conditions in his analysis. Resnik argues that TF bans may meet the effectiveness, proportionality, and public justification conditions, but may fail to meet the necessity and least infringement conditions (2010, 31). Resnik defines necessity and least infringement as follows:

Necessity: there must be substantial scientific evidence that the policy is necessary to achieve the public health goal (30).

Least infringement: the policy must impose the least restrictions on freedom necessary to promote the public health goals (30).

Before continuing, note that Resnik’s definition of least infringement is not an accurate representation of the condition as stated by Childress et al. Resnik refers only to restrictions on freedom. However, recall that the Childress et al. framework requires least infringement of “general moral considerations” (2002, 173)—i.e. “clusters of moral concepts and norms that are variously called values, principles, or rules” (171, 173). Liberty is only one such consideration: others include “producing benefits,” “distributing benefits and burdens fairly,” “protecting privacy and confidentiality,” and so on (171–172). Resnik thus uses least infringement in a narrower sense than is intended by Childress et al.—i.e. as the “least restrictive alternative” condition posited in other public health ethics frameworks, such as in Gostin (2005).

This point aside, the following discussion aims to determine whether the rest of Resnik’s evaluation of TF bans employs the necessity and least infringement conditions (as stated by Childress et al.) correctly. Resnik argues that TF bans may fail to satisfy necessity “because a combination of other policies, such as education and mandatory labeling, may be equally effective at achieving public health goals” (2010, 31)—although this is an open empirical question, as he acknowledges. It is unclear whether Resnik is applying the necessity condition in the way intended by Childress et al. One interpretation is that Resnik argues simply that TF bans may not be the *only way* to achieve the given public health goals. On this interpretation, his argument is as follows: since there may be alternatives to TF bans, TF bans may not be *necessary*, and thus TF bans may not pass the necessity condition. However, if this is Resnik’s argument, then he is working with the wrong conception of the necessity condition, and thus fails to show that TF bans may not pass the necessity condition. As argued above, the necessity condition cannot plausibly require that an intervention be the *only possible way* to realise the goal. Instead, it must mean that the costs imposed by the intervention are necessary, i.e. that the costs cannot be avoided because there is no less costly alternative way to achieve the public health goal in question. The other interpretation of Resnik’s argument is that he *does* employ this correct conception of necessity. On this interpretation, his argument implicitly contains the premise that the alternative policies (i.e., such as education and mandatory labelling) are *morally preferable* because they are less infringing of moral considerations than TF bans—though this is not explicitly said by Resnik. If this is Resnik’s intention, then the argument is instead that TF bans may fail to meet necessity because the public health end may be realised via less morally costly interventions.

In arguing that trans fat bans may not meet least infringement, Resnik ranks a number of different strategies from least restrictive of liberty to most restrictive. He argues that education is the least restrictive alternative, while mandatory food labelling, taxation, and food safety and quality standards are increasingly restrictive. Finally, bans on particular food items are the “most restrictive methods of promoting public health,” according to Resnik, because “bans prevent people from making some types of dietary choices and they prevent food producers from selling particular types of foods” (31).

## Confusion relating to necessity

Wilson and Dawson (2010) object to Resnik’s argument, arguing that he misuses both the necessity and the least infringement conditions. First, they critique Resnik’s employment of the necessary condition, as follows:

The necessity criterion simply asks us to consider whether there are other ways of regulating a particular public health risk that have a better balance of moral benefits over moral costs. Costs to liberty are only one of the relevant factors. The very probable large costs to human life through waiting to see whether education and labeling policies are effective ways of reducing trans fat intake is also a relevant factor. Resnik provides no reason to think that the moral costs associated with these very probable deaths are smaller than those associated with the infringement of liberty (Wilson and Dawson 2010, 35–36).

Wilson and Dawson argue that Resnik does not consider all of the moral costs and benefits that are relevant to whether or not TF bans are implemented. They are right about this: as mentioned above, Resnik does indeed consider only liberty restrictions, rather than broader infringements of general moral considerations.

While Wilson and Dawson are thus partly right in their criticism, they themselves hold a mistaken account of Childress et al.’s necessity condition. Necessity does not require us to “consider whether there are other ways of regulating a particular public health risk that have a *better balance of moral benefits over moral costs*” (Wilson and Dawson 2010, 35–36, emphasis added), but instead to simply pick the least infringing alternative *of equally effective interventions*. To illustrate this difference with an example about infectious disease control, suppose education campaigns are able to realise a total of 10 benefits at a cost–benefit ratio of 1:10 (i.e. 10 benefits for every 1 cost), while quarantine measures are able to realise a total of 20 benefits at a ratio of 10:20 (i.e. 20 benefits for every 10 costs). In this hypothetical case, education campaigns have a better *ratio* or *balance* of costs and benefits than quarantine, but quarantine measures produce twice the amount of benefits, i.e. are twice as effective. Since the Childress et al. necessity condition requires the least infringing alternative of *equally effective* interventions be chosen, the condition would not require that education campaigns be prioritised over quarantine measures in this case, because they are not equally effective.

A mistaken conception of necessity also leads Wilson and Dawson to equivocate between two kinds of moral costs. One type of cost that they argue *should* be counted by Resnik is “[t]he very probable large costs to human life through waiting to see whether education and labeling policies are effective…” (Wilson and Dawson 2010, 36). However, this cost—while important—is not one that is counted by the necessity condition as stated by Childress et al. The necessity condition is supposed to count what we call ‘infringement costs,’ i.e. the costs that are caused by the intervention itself when it is implemented; but necessity is not concerned with a distinct kind of cost, ‘foregone benefits,’ i.e. the costs that occur when an effective intervention is *not* implemented and when the alternative interventions are not equally effective.^3^^,^ 4 Foregone benefits are not counted because necessity (as stated by Childress et al.) applies only in cases of equally effective interventions, and there simply cannot be foregone public health benefits when one intervention is chosen over another that is equally effective. Considering foregone benefits would be an *additional* requirement that is not built into the framework. Thus, other than their first argument—which is sound (as discussed above)—Wilson and Dawson fail to show that Resnik applies necessity in a way that is inconsistent with necessity as conceived by Childress et al., because Wilson and Dawson themselves appear to misunderstand Childress et al.’s necessity principle.

### Limitations of the necessity condition

The debate between Wilson and Dawson (2010) and Resnik (2010) reveals two limitations of the necessity condition. First, necessity cannot guide action when comparing interventions that are not equally effective, as mentioned above. This is not to say that the necessity condition is incorrect; rather, it can be applied in only a limited set of cases. In the cases in which it applies, i.e. when choosing between equally effective interventions, the necessity condition is still surely correct that the least infringing alternative is the morally best alternative, other things being equal. We take this to be self-evidently true.^5^

Second, the above debate reveals that necessity cannot guide action in cases of empirical uncertainty. It would be unclear whether the necessity condition would apply in a given case if there is uncertainty whether alternative measures *are* equally effective. For example, Resnik’s argument is that it cannot be determined whether TF bans are necessary, since there is a lack of evidence about the effectiveness of alternative measures, which may or may not turn out to be sufficiently effective to realise the goal. His conclusion is that TF bans should not be implemented until there is more evidence about the effectiveness of alternatives. But, as revealed by Wilson and Dawson, this approach delays an important public health response to the obesity problem, and thus results in high costs in terms of foregone benefits. How should we proceed in such a case? According to Childress et al., the following approach should be taken when applying the necessity condition in contexts of empirical uncertainty:

Proponents of the forcible strategy have the burden of moral proof. This means that the proponents must have a good faith belief, for which they can give supportable reasons, that a coercive approach is necessary. In many contexts, this condition does not require that proponents provide empirical evidence by actually trying the alternative measures and demonstrating their failure (Childress et al. 2002, 173).

While this suggests that Resnik may follow the framework correctly in his argument, the approach prescribed by the framework itself is unsatisfactory. The claim that proponents of the infringing strategy have the burden of proof is dubious (see Koplin and Selgelid (2015) for a critical evaluation of the concept of a burden of proof in bioethics). Childress et al.’s approach seems to be motivated by the view that when an intervention is expected to infringe moral considerations, and thus create infringement costs, a justification for those costs must be offered. However, this view loses some initial appeal when the scenario is reframed in terms of foregone benefits: i.e., when an intervention is to be delayed due to empirical uncertainty about the effectiveness of alternative measures, a justification for the foregone benefits caused by the delay arguably must be offered.

There is, moreover, another reason to doubt that proponents of the infringing strategy should bear the burden of proof. Since the necessity condition is supposed to apply in cases of equally effective interventions, those undertaking the evaluation should presumably have an obligation to demonstrate that any alternative interventions would be equally effective. In other words, it seems problematic for the public health ethicist to simply claim that alternatives measures (e.g. education campaigns) *might* be just as effective as a morally costly intervention (e.g. TF bans), without providing any evidence for their claim. Doing so could cause the unwarranted delay of important public health interventions. It would thus be highly problematic for the Childress et al. framework to hold that one party or the other bears the burden of proof. Instead, the framework should remain neutral and provide guidance on how to proceed in contexts of empirical uncertainty. While further attention is required to determine how the framework should provide guidance in contexts of empirical uncertainty, a possible approach is outlined in the final section of this paper.

## Confusion relating to least infringement

Wilson and Dawson’s refutation of Resnik’s application of the least infringement condition is as follows:

[I]nvoking the “principle of the least restrictive alternative”^6^ in public health regulation seems to be either almost entirely superfluous, or to involve an illicit form of double counting. If it means that we should adopt the least restrictive alternative out of the ones that have already been singled out as offering the best balance of moral benefits over moral costs, then presumably it can only operate as a tie breaker, and so will be unable to do the work Resnik requires. But if it is supposed to play a substantive role (as Resnik seems to imagine it will), then it seems to be clearly illicit, given that liberty has already been weighed in the balance against the other goods at the stage of working out which policies are proportional and necessary. Liberty is an important value, but it is not so important that it ought to be counted multiple times (36).

Wilson and Dawson’s objection to Resnik’s use of the least infringement condition takes the form of a dilemma: either its use is superfluous because it breaks ties in favour of liberty, or involves an illicit form of double counting. We argue that neither of these charges is legitimate.

First, Wilson and Dawson’s double counting objection is that Resnik seems to weigh liberty in each of the proportionality, necessity and least infringement stages of evaluation, but they take it to be problematic to weigh liberty more than once. However, the Childress et al. framework does not work by counting the number of times a moral value is weighed. Instead, the framework involves determining whether a policy satisfies each of the justificatory conditions. It is surely necessary to weigh liberty against other values in a number of these stages: it matters whether the benefits of trans fat bans are proportionate to the costs of the bans, including costs to liberty (i.e. proportionality); whether the infringements caused by trans fat bans are necessary to achieve the goal (i.e. necessity); and whether trans fat bans infringe moral considerations to the least possible degree (i.e. least infringement).^7^ Given that these are *necessary* conditions, the intervention is ethically acceptable only if it satisfies every condition. If it *fails* to satisfy every condition, it is simply impermissible, and the number of conditions it passes does not change this outcome. To illustrate, suppose three of the many necessary conditions to keep a plant alive are that the plant receives sufficient water, the plant receives sufficient sunlight, and the plant is not drowned in water. It is not better to fail ‘only’ one condition than two, because in either case the plant dies. Moreover, it makes no sense to say that since water is counted in two conditions, while sunlight is counted in one, water is being given too much importance in determining whether the plant survives. Such reasoning shows that it is not problematic for liberty to be weighed against other values multiple times when evaluating a policy; in fact, doing so seems necessary.

The other horn of the dilemma is that if least infringement is used as a tiebreaker, it is superfluous. Wilson and Dawson rightly argue that least infringement would be a tiebreaker if it is used to prioritise “the least restrictive alternative out of the ones that have already been singled out as offering the best balance of moral benefits over moral costs” (36). To illustrate, suppose interventions A and B are equally effective and involve equal amounts of moral cost, but A infringes only liberty, while B infringes only justice. If least infringement requires that we adopt the least restrictive (i.e. least infringing of liberty) alternative of equally balanced interventions, as Wilson and Dawson say it might, then it would require that the tie between A and B be broken in favour of B, because B is the alternative that is least infringing of liberty. Wilson and Dawson are right that such a tiebreaker condition would be problematic because it involves an arbitrary bias against liberty infringement. If A’s infringement of liberty is *equally morally costly* as B’s infringement of justice, then there presumably can be no reason to choose one intervention over the other, and doing so is therefore arbitrary.

While this reasoning is sound, the least infringement condition as stated by Childress et al. does *not* apply in such tiebreaker cases. The least infringement condition applies only in comparisons of equally effective interventions with the aim to find the least infringing (i.e. least morally costly) alternative. If the interventions in question are also *equally morally costly*, then there can be no *least costly* alternative. Moreover, the tiebreaker objection makes sense only if least infringement is taken to be concerned only with *liberty restriction*. In the above example where interventions A and B involve equal costs and benefits, least *restriction* (i.e. concerned only with liberty costs) would prioritise B over A because B infringes only justice, while A infringes liberty. However, as discussed, least infringement as stated by Childress et al. is concerned not only with liberty restriction, but with infringements of moral considerations generally. Least infringement thus *cannot* break ties in cases of equally effective and equally costly interventions, because in such cases it is logically impossible for one intervention to be less infringing than another when all costs are considered. For these reasons, Wilson and Dawson fail to show that least infringement may be an arbitrary tiebreaker.

While least infringement is thus not superfluous in the above sense, we argue it *is* superfluous, but for another reason: least infringement is logically equivalent to necessity, as argued above. It is therefore redundant to list both necessity and least infringement in the framework. Aside from this unnecessary redundancy, listing both conditions is harmless in itself because listing the same necessary condition multiple times cannot change the output of the framework. However, listing both necessity and least infringement leaves more room for confusion and error, as seen in the debate between Resnik and Wilson and Dawson. Since necessity and least infringement are different ways of describing the same condition, only one principle is needed to state that condition. While it does not matter what the principle is called, it is referred to as least infringement in the remainder of this article.

## Conceptions of least infringement in public health ethics

There are a number of different versions of least infringement principles currently in the literature. Some public health ethics frameworks, such as Gostin (2005) and Upshur (2002), include some version of a *least restrictive alternative* principle. A least restrictive alternative principle would differ from Childress et al.’s least infringement principle insofar as the former is concerned only with the infringement of liberty, while the latter refers to “general moral considerations” (Childress et al. 2002, 173), including any relevant moral values such as liberty, privacy, justice and so on. Least *infringement* is thus a broad requirement that implies least *restriction* (other things being equal), which is a narrower corollary requirement that focuses on costs to liberty. The two are not logically equivalent: the least infringement requirement implies the least restriction requirement, but not vice versa. In other words, the requirement that an intervention be the least infringing alternative implies that the intervention be the least restrictive alternative (among those that are equally effective, and equally costly of other moral infringements besides liberty, i.e. all else being equal). This reasoning can be used to show that there are corollaries of least infringement for each kind of moral cost. For example, the requirement that an intervention be the least infringing alternative also implies that the intervention be the alternative that infringes *privacy* to the least degree (all else being equal), and also that it be the alternative that infringes *justice* to the least degree (all else being equal), and so on.

Another principle that is similar to the least infringement condition is included in Kass’s (2001) public health ethics framework, as one of six steps to evaluate interventions. The idea is expressed in two senses: minimizing burdens, and seeking alternative approaches.

[W]e are ethically required to determine whether the program could be modified in ways that minimize the burdens while not greatly reducing the program’s efficacy (1780).

If 2 options exist to address a public health problem, we are required, ethically, to choose the approach that poses fewer risks to other moral claims, such as liberty, privacy, opportunity, and justice, assuming benefits are not significantly reduced (1780).

Kass’s conception of least infringement differs from the least *restriction* principles of Gostin and Upshur: as discussed, the latter two are concerned only with liberty, while Kass refers to a number of moral values. Kass’s requirements to minimize burdens and seek alternative approaches thus seem to amount to a requirement that is more similar to the one posed by Childress et al.’s least infringement condition.^8^

## Narrowing the scope of least infringement

The least infringement principle—as stated by Childress et al. (2002)—seems to consist of two components: an evaluative one and a prescriptive one. The evaluation is that the least infringing intervention of equally effective alternatives is the *morally best* intervention—assuming all else is equal. This evaluation is self-evidently correct. What are its normative implications? When this evaluation is considered on its own—i.e., in isolation from the rest of the Childress et al. framework—it does not tell us which specific course of action should be taken. For example, suppose there are three equally effective public health interventions available—A, B, and C—and A is determined to be the least infringing alternative. The evaluation that A is the morally best intervention does not on its own tell us whether only A should be implemented, or instead whether some combination of A, B and C is preferable. It could be that while A is the least infringing alternative, B might still be only slightly more infringing than A, and thus may be morally acceptable. If it were the case that implementing both A and B would yield greater net benefits than implementing only A, for example, then it may be morally acceptable to implement both. In contrast, if implementing both A and B would *not* yield greater net benefits than implementing only A, then only the least infringing alternative—in this case A—should be implemented.

However, the Childress et al. (2002) framework might entail the prescription that when there are two or more equally effective alternatives available, *only* the least infringing alternative is ethically justified. Whether or not the framework does entail this prescription is unclear. Childress et al. say that interventions should be the least infringing alternative to “realize the public health goal that is sought” (173). This is ambiguous because it is unclear precisely what is meant by the “public health goal.” Let us distinguish between ‘goals’ and ‘subgoals’ of interventions. The overarching goal of public health interventions is typically to protect and/or promote public health, while the subgoal of an intervention pertains to the specific means by which the overarching goal is realised. Different interventions may share the same overarching goal but have different subgoals. For example, there may be multiple interventions available to protect the public from an infectious disease outbreak, but a quarantine measure has as its subgoal to prevent the spread of the disease, while an intervention to provide more medications includes the subgoal of reducing harm to affected individuals. If two interventions aim to realise exactly the same subgoal—that is, if they aim to bring about exactly the same state of affairs—then no additional benefit would be produced by implementing both interventions than by implementing only one of them. In contrast, if multiple interventions have (or include) different subgoals, then implementing multiple interventions *would* bring about more benefit than implementing only one of them. That is, it would be better for the health of the public not only to prevent the spread of disease via quarantine, but also to supply more medications to affected individuals.

In light of these considerations, if Childress et al. intend to say that interventions must be the least infringing alternative to realise the *overarching* goal, then the framework does entail that only *one* intervention—the least infringing alternative—can be implemented, even if implementing multiple interventions could realise greater net benefit by achieving different subgoals. However, if Childress et al. intend to say that interventions must be the least infringing alternative to realise a particular *subgoal*, then the framework does allow the implementation of multiple interventions that have different subgoals. It is ultimately unimportant which of these approaches is intended by Childress et al. because it seems that the correct approach must be to say that interventions should be the least infringing alternative to realise a particular subgoal. The least infringement principle would be implausible if it were to prohibit the implementation of multiple (otherwise ethically justified) interventions that together bring about greater net benefit than implementing only one of them. Thus, the least infringement principle should prescriptively apply only in cases where two or more interventions are available to achieve the same particular subgoal.^9^

## Conclusions and the way forward

The public health ethics framework proposed by Childress et al. (2002) is valuable as it points to important moral values. However, we have argued the framework is problematic in numerous ways. We have demonstrated that necessity and least infringement are logically equivalent, and thus redundantly state the same condition. Listing both in the framework, as though they were distinct/separate conditions, may be unnecessarily confusing for practitioners or policymakers, and collapsing the two principles into one may thus be helpful in practice. However, even with the refinements advocated above in this article, there are still problematic aspects of the framework that may limit its usefulness in practice. We have identified three limitations of the least infringement condition as stated by Childress et al.: (1) it can guide action only in cases of equally effective interventions; (2) it does not provide guidance in contexts of empirical uncertainty; and (3) it can guide action only in cases where only one intervention is to be implemented to achieve one particular subgoal. Moreover, we contend that the framework as a whole is problematic due to it being composed of necessary conditions.^10^^,^ 11 One potentially promising approach is, rather than requiring that interventions satisfy a number of necessary conditions, to evaluate the *degree to which* interventions achieve various ethical desiderata—i.e., scalar dimensions upon which interventions could fare better or worse. A scalar version of the Childress et al. framework would hold that, other things being equal, public health interventions are ethically acceptable *to the degree that* they are effective, proportional, necessary/least infringing, and publicly justified.^12^ Rather than determining whether any given intervention is ethically acceptable or not, such a framework would locate any given intervention on a scale ranging from those that are most ethically acceptable (i.e., those that fare best on all dimensions) to those that are most ethically unacceptable (i.e., those that fare worst on all dimensions). Such a framework would avoid asking either-or questions about whether particular conditions are satisfied or not, and it could (contrary to a framework consisting of necessary conditions) allow strong performance on some dimension(s) to compensate for suboptimal performance on others.

Among other benefits, a scalar approach to public health ethics may provide better guidance in contexts of empirical uncertainty—for example, it could account for our *confidence* that an intervention would cause a low magnitude of moral costs (which in practice will often be a matter of degree), rather than asking *whether or not* an intervention passes the least infringement condition (which might often be unknowable). Further development of a scalar approach to public health ethics frameworks is thus an important area for further research. It is beyond the scope of this paper to discuss such an approach in more detail, but see e.g. Selgelid (2009) and Selgelid (2016) for additional discussion of the motivation for such a shift and further indication of what a scalar framework for public health ethics might look like.

## Footnotes

1 The discussion of the least infringement principle is worded slightly differently in a later paper by Childress and Gaare Bernheim (2008, 160).

2 A similar view is discussed briefly by Nobis and Gardner in response to Resnik’s (2010) statement of the necessity and least infringement principles: ‘[s]ince these two conditions sound similar, we propose to treat them as one condition’ (Nobis and Gardner 2010, 40).

3 We use the term ‘foregone benefits,’ rather than the more common term ‘opportunity costs,’ in cases where costs occur when an effective intervention is not implemented and when the alternatives are not equally effective. We would use the term ‘opportunity costs’ in cases where other effective alternatives are foregone when an intervention *is* implemented, i.e. if there is a limit to the number of interventions that can be implemented.

4 Unless explicitly referring to ‘foregone benefits,’ by the term ‘cost’ we refer only to infringement costs, i.e. the costs related to the infringement of moral considerations caused by interventions.

5 While we say here that necessity applies in cases of comparisons between equally effective interventions, we argue in a subsequent section of the paper that necessity applies only in an even narrower set of cases, i.e. cases in which only one intervention is to be implemented to achieve one specific subgoal.

6 Though Wilson and Dawson refer to the “principle of the least restrictive alternative,” it is clear that they are discussing Resnik’s employment of the “least infringement condition.”

7 Although, given that necessity and least infringement are logically equivalent, the question of whether the intervention’s infringements are necessary is the same as the question of whether the intervention infringes moral considerations to the least possible degree.

8 The quotes of Kass above, however, reveal that her principle is less limited to adjudication between equally effective/beneficial alternatives.

9 The usefulness of the least infringement principle in practice partly depends on the frequency with which such situations arise.

10 The move to a scalar framework that we propose is partly motivated by feedback we have repeatedly received that frameworks consisting of necessary conditions are not very helpful in practice. That is, whether or not an intervention will pass a given necessary condition is a binary question, yet answering such a question—i.e., by locating a threshold that determines whether the condition is passed or failed—is often intractable. Consequently, practitioners and policymakers often say that they cannot get past the first condition when using a framework consisting of necessary conditions—i.e., because it is not easy to determine whether the condition in question has been met or not.

11 A further motivation for this move is that frameworks consisting of necessary conditions ultimately amount to showing that a number of boxes can be ticked. By requiring instead that judgments be made regarding the *degree* to which various ethical desiderata are achieved, a scalar framework would focus practitioners’ attention on key values that matter, and the degree to which they might (or are expected to) be promoted or compromised by alternatives under consideration, rather than on checklists.

12 The point here is that—rather than being either-or affairs—effectiveness, proportionality, necessity/least infringement, and public justification are each dimensions along which performance can be better or worse and/or (at least) for which our confidence about performance can be stronger or weaker.
